# Prevalence of urinary schistosomiasis and associated risk factors among Abobo Primary School children in Gambella Regional State, southwestern Ethiopia: a cross sectional study

**DOI:** 10.1186/s13071-015-0822-5

**Published:** 2015-04-10

**Authors:** Shashie Geleta, Agersew Alemu, Sisay Getie, Zeleke Mekonnen, Berhanu Erko

**Affiliations:** Gambella Teachers’ Education and Health Sciences College, Gambella, Ethiopia; Department of Medical Parasitology, School of Biomedical and Laboratory Sciences, University of Gondar, Gondar, Ethiopia; Department of Medical Laboratory Sciences and Pathology, Jimma University, Jimma, Ethiopia; Aklilu Lemma Institute of Pathobiology, Addis Ababa University, Addis Ababa, Ethiopia

**Keywords:** Urinary schistosomiasis, Risk factors, School children, Abobo, Gambella, Ethiopia

## Abstract

**Background:**

In Ethiopia, urinary schistosomiasis caused by *Schistosoma haematobium* has been known to be endemic in several lowland areas of the country where it causes considerable public health problems, mainly among school-age children. However, information on recent magnitude and risk factors of the disease is lacking, particularly for Gambella area. Therefore, this study aimed to assess the prevalence of urinary schistosomiasis and associated risk factors among Abobo Primary School children in Gambella, southwestern Ethiopia.

**Methods:**

A cross-sectional study involving 304 school children was conducted in Abobo Primary School, Gambella Regional State, southwestern Ethiopia, from February to June 2014. Ten ml of urine sample was collected from each study participant and processed for microscopic examination by the urine filtration method; egg load for positive individuals was determined per 10 ml of urine. Data on socio-demographic characteristics and risk factors were collected using an interview-based questionnaire. The data were entered into and analyzed with SPSS version 20. Logistic regression and odds ratio were used to measure association and strength between variables, respectively. P-value < 0.05 at 95% CI was considered as statistically significant.

**Results:**

The prevalence of urinary schistosomiasis was 35.9% (109/ 304) with a mean egg intensity of 8.76 per 10 ml of urine. Being male [AOR (95%CI) = 2.15(1.31, 3.52)], having father as a farmer [AOR (95%CI) = 1.96(1.19, 3.22)] and children living apart from parents [AOR (95% CI): 3.09 (1.14, 8.4)] were significantly associated with urinary schistosomiasis.

**Conclusion:**

The present study area in Gambella Regional State, southwestern Ethiopia, represents moderate-risk community for urinary schistosomiasis. Sex, father’s occupation and living apart from parents were found to be associated with infection. Treatment of all school-age children and fishermen is required once every 2 years until the prevalence of infection falls below the level of public health importance. It is also recommended to complement praziquantel treatment with supplementary measures such as provision of sanitation facilities and health education.

## Background

Human schistosomiasis is a chronic disease caused by the blood flukes belonging to the genus *Schistosoma*. The main disease causing schistosome species are *Schistosoma haematobium (S. haematobium), S. mansoni, S. japonicum, S. mekongi and S. intercalatum* [1]. Schistosomiasis is estimated to affect 249 million people worldwide, of which at least 224 million affected people live in sub-Saharan Africa [2]. It ranks second only to malaria as the most common parasitic disease, killing an estimated 280,000 people each year in the African region alone [3].

*S. haematobium* is the aetiologic agent of urinary schistosomiasis and it is most prevalent in Africa [4]. In sub-Saharan Africa, *S. haematobium* infection is estimated to cause 70, 32, 18 and 10 million cases of hematutria, dysuria, bladder-wall pathology and major hydronephrosis, respectively [5]. The infection is also responsible for nutritional deficiencies and growth retardation [6], adverse effects on cognitive development [7], as well as for decreasing physical activity, school performance, and work capacity and productivity [6].

Transmission of urinary schistosomiasis is dependent on availability of specific snail hosts and human activities with water contacts [8]. Therefore, the risk and reemergence of urinary schistosomiasis is attributed to the range of snail habitats promoted by water development schemes such as dam construction [9]. On the other hand, school age children were thought to have frequent water contact that would make them more vulnerable to schistosomiasis, and hence this age group would be associated more frequently with schistosomiasis problems [10,11].

In Ethiopia, both *S. haematobium and S. mansoni* are endemic, with an estimated 4 million people infected and 30–35 million being at risk of infection [12,13]. An infection rate of urinary schistosomiasis as high as 62.7%, was reported among children younger than 14 years of age in certain parts of the country. However, the distribution of urinary schistosomiasis in Ethiopia is highly focal and is limited to lowland areas. In addition, the development of irrigation schemes in different areas of the country has contributed to spread of the disease [12].

In order to put in place appropriate interventions against schistosomiasis, information on the distribution and associated risk factors of the disease in different transmission settings is a prerequisite. This study, therefore, aimed to determine the prevalence and associated risk factors of *S. haematobium* infection among primary school children at Abobo District, Gambella, southwestern Ethiopia.

## Methods

### Study area

**Figure 1:**
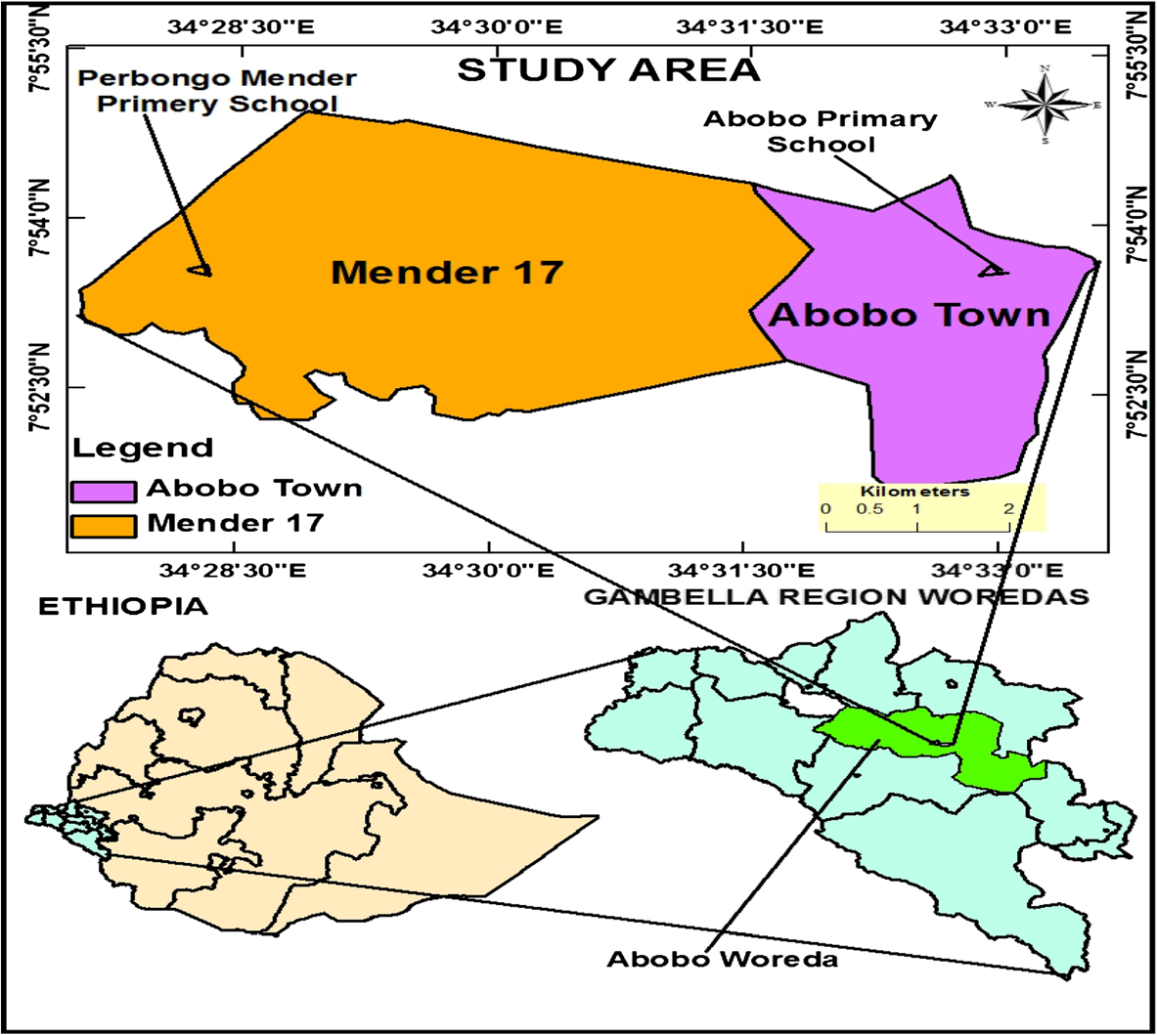
**Map of the study area, Abobo District, Gambella Regional State, Ethiopia.**

The study was conducted at Perbongo Mender 5/6 and among Abobo Primary Schools in Abobo Town, Abobo District, Gambella, Ethiopia (Figure 1). Abobo is located at 7° 51′ 0″ North, 34° 33′ 0″ East, and 45 km south of Gambella Town and 822 km southwest of Addis Ababa, Ethiopia. The people in the community are engaged mainly in agricultural and fishing activities. There is a large river called ‘Alwero’ across Abobo Town where Alwero dam is constructed and children rely on it for swimming and fishing.

### Study design, period and participants

A cross-sectional study was carried out from February 2014 to June 2014. Study participants were recruited voluntarily from Perbongo Mender 5/6 and Abobo Primary Schools in Abobo Town, Gambella.

### Inclusion and exclusion criteria

Primary school children in the 7–14 years age group, attending Perbongo Mender 5/6 and Abobo Primary Schools during the study period were enrolled.

### Exclusion criteria

Primary school children who took medication for schistosomiasis three weeks prior to and during the data collection and children who were seriously ill during data collection were excluded.

### Sample size

The required sample size was determined using single population proportion formula and assuming a 24.54% proportion of urinary schistosomiasis [14], 5% level of significance (α = 0.05), 95% confidence interval and 5% margin of error (D = 0.05).

After computation and adding 10% for non-response rate, the final sample size was 313. Thus, a total of 313 primary school children participated in the study.

### Sampling technique

Perbongo Mender 5/6 and Abobo Primary Schools were selected randomly from ten primary schools in Abobo District. Sample size was proportionally allocated to each school and each class of participating schools. The student registration book was used as a sampling frame and study participants were selected using a simple random sampling technique. Accordingly, 223 and 90 study participants were recruited from Perbongo Mender 5/6 and Abobo Primary Schools, respectively.

### Data collection procedures

#### Socio-demography and related factors

Interview based questionnaire prepared in English and then translated into Agnuak and translated back to English was used to collect socio-demographic and associated risk factor data for urinary schistosomiasis.

### Urine sample collection

About 10 ml of terminal urine sample was collected from each study participant in clean labelled plastic containers. Then, 0.2 ml of 37% formalin was added to the urine sample as a preservative. The urine sample was collected between 10 AM and 2 PM to obtain high *S. haematobium* egg load [15].

### Microscopic diagnosis of *Schistosoma haematobium* infection

A standard filtration technique was used to diagnose and quantify ova of *S. haematobium*. Mixed 10 ml of urine was allowed to pass through 13 mm diameter and 12 μm pore size of nylon mesh filter (Costar Corporation, USA) supported with plastic syringe. The filter containing the residues including *S. haematobium* eggs was removed and placed on a clean microscopic slide to be examined under a middle power objective. After examining the whole field, microscopic slides containing eggs of *S. haematobium* were recorded as positive while absence of eggs was taken as negative. Intensity of infection was determined for positive samples and recorded as number of eggs per 10 ml of urine. The intensity was classified as light infection (less than 50 eggs/10 ml of urine) and heavy infection (more than 50 eggs/10 ml of urine) [15].

### Quality control

Ten percent of the urine samples were re-checked by an experienced laboratory technician. Data was collected by the first author and other trained personnel and its completeness was checked before the study subject left**.** Any error appearing during data entry into the computer was re-checked against the original hard copy.

### Data analysis

The data were entered into and analyzed using SPSS version 20. Data summary was made using descriptive statistics. Chi square, bivariate and multivariable logistic regressions were used to determine the association between dependent and independent variables. Odds ratio with 95% CI was used to measure strength of association between variables. Statistical significance was considered at 95% CI and P-value less than 0.05.

### Ethical consideration

The protocol of the study was reviewed and approved (Reference Number: SBMLS/525/06, 2014) by Research and Ethical Committee of School of Biomedical and Laboratory Sciences, College of Medicine and Health Sciences, University of Gondar, Gondar, Ethiopia. Permission to conduct the study was obtained from Gambella Regional Health Bureau and Gambella Regional Educational Bureau. Informed assent and consent was obtained from children and parent/guardian of the children, respectively. All the information obtained from each study participant was kept confidential. Children found positive for *S. haematobium* eggs were treated with praziquantel using a single oral dose of 40 mg/kg body weight at Abobo Health Center by a physician.

## Results

### Socio-demographic characteristics of study participant

A total of 313 primary school children were recruited and 304 were included in the analysis, of which 155 (51%) and 149 (49%) of them were males and females, respectively. The mean age (SD) of the study participants was 11.37 (2.32) years. Non-farmer (52.3%) and housewife (64.4%) were the major occupation for fathers’ and mothers’ of study participants, respectively. Among study participants’ parents, 165 (58.9%) of fathers and 140 (47.6%) of mothers were able to read and write (Table 1).

**Table 1:** **Socio-demographic characteristics of study participants, Abobo, southwestern Ethiopia, 2014**

**Variables**	**Frequency**	**Percentage**
**Age**		
7-9	67	22.0
10-12	99	32.6
13-14	138	45.4
**Sex**		
Male	155	51
Female	149	49
**Educational level**		
1-4	131	43.1
5-8	173	56.9
**Fathers’ occupation**		
Farmer	145	47.7
Non farmer	159	52.3
**Mothers’ occupation**		
House wife	190	62.5
Government employee	28	9.2
Daily labourer	30	9.9
Other	56	18.4
**Fathers’ educational status**		
Does not read and write	66	21.7
Able to read and write	165	54.3
Secondary school completed	54	17.8
Expert level	19	6.3
**Mothers’ educational status**		
Does not read and write	143	47.0
Able to read and write	140	46.1
Secondary school completed	21	6.9
**Total**	**304**	**100%**

### Prevalence and intensity of urinary schistosomiasis

In this study, the prevalence of urinary schistosomiasis was 35.9% with a mean (SD) intensity of 8.76 (14.51) per 10 ml of urine. Among infected study participants, 106 (97.2%) presented with light intensity of urinary schistosomiasis and 3 (2.8%) with heavy intensity. The frequency and mean intensity of urinary schistosomiasis was higher among males [61.5% (χ ^2^ = 7.47, p < 0.008)] and 9.36 per 10 ml of urine) than females (38.5% and 7.81per 10 ml). On the other hand, higher frequency of urinary schistosomiasis was identified among children in the 13 to 14 years age group (39.1%) than the 7 to 9 years age group (15.6%), and the 10 to 12 years age group (34.9%), (χ ^2^ = 4.12, p < 0.127). However, the mean egg count was higher among children in the 10 to 12 years age group (11.18 per 10 ml). Nevertheless, there was weak linear relationship (r = −0.007) between age of children and egg count of urinary schistosomiasis. Overall, there was no statistically significant association between mean egg count of urinary schistosomiasis and sex, age group, distance from the river, use of river for drinking and washing, and swimming habit (data not shown). Higher frequency of urinary schistosomiasis was identified among children with farmer as father’s occupation (56.0%) (χ ^2^ = 4.65, p < 0.032) and housewife as mother’s occupation (64.8%) (χ ^2^ = 0.866, p < 0.834). Children with a habit of swimming accounted for 91.7% ((χ ^2^ = 0.496, p < 0.552) of the total urinary schistosomiasis prevalence.

### Factors associated with urinary schistosomiasis

In this study, 274 (90.1%) of the study participants had a habit of swimming in rivers. However, only 60 (21.9%) of them experienced regular swimming. On the other hand, 213 (70.1%) of the study participants required less than 30 minutes before reaching the river dam and 91 (29.9%) of them used it for washing clothes and bathing. Of the study participants, 168 (56.6%) had heard about urinary schistosomiasis and school was the source of information for 76 (44.2%) of them. In spite of this, 52 (30.2%) of them didn’t know any ways of prevention or control against urinary schistosomiasis (Table 2).

**Table 2:** **Frequency of risk factors associated with urinary schistosomiasis, Abobo, southwestern Ethiopia, 2014**

**Variables**	**Frequency**	**Percentage**
**Swimming habit**		
Yes	274	90.1
No	30	9.9
**Swimming regularity**		
Some times	214	78.1
Regularly	60	21.9
**Living with parent**		
Yes	284	94
No	18	6
**Distance from river**		
<30min	213	70.1
>=30min	91	29.9
**Water source for washing and bathing**		
Alwero Dam	91	29.9
Other	213	70.1
**Heard about urinary schistosomiasis**		
Yes	168	56.6
No	136	43.4
**Total**	304	100
**Knowledge about urinary schistosomiasis among the total 168 participants who had heard about urinary schistosomiasis**		
**Information source**		
School	76	45.2
Home	46	27.4
Other	46	27.4
**Symptom of urinary schistosomiasis**		
Abdominal pain	16	9.5
Dysuria	5	3.0
Bloody urine	121	72.0
Weakness	4	2.4
Do not know	22	13.1
**Parasite penetrate skin**		
Yes	48	28.6
No	120	71.4
**Know the vector**		
Yes	30	17.9
No	138	82.1
**Have prevention**		
Yes	142	69.0
No	10	14.9
Do not know	16	16.1
**Have treatment**		
Yes	142	84.5
No	10	6.0
Do not know	16	9.5
**Total**	168	100

### Multivariate analysis of factors associated with urinary schistosomiasis

In the bivariate analysis, the 13–14 years of age group [COR (95% CI): 1.89 (1.89, 3.61)], male [COR (95% CI): 1.94(1.2, 3.12) and farmer as father’s occupation [COR (95% CI): 1.68(1.05, 2.69)] were significantly associated with urinary schistosomiasis. In the multivariate logistic regression analysis, only sex, father’s occupation and living with parent had statistically significant association with urinary schistosomiasis. Males were two times more likely to be infected with *S. haematobium* than females, AOR: 2.13 (95% CI: 1.3, 3.5) (Tables 3 and 4).

**Table 3:** **Multivariate analysis of variables associated with frequency of urinary schistosomiasis among study participants, Abobo, southwestern Ethiopia, 2014**

**Variables**	**Frequency of urinary schistosomiasis**			**COR (95%CI)**	**AOR (95% CI)**
	**Sub-total (%)**	**Positive (%)**	**Negative (%)**		
**Age**					
7-9	67 (100)	17 (25.4)	50 (74.6)	1	-
10-12	99 (100)	38 (38.4)	61 (61.6)	1.83 (0.93,3.63)	
13-14	138 (100)	54 (39.1)	84 (60.1)	1.89 (1.89,3.61)	
**Sex**					
Male	155 (100)	67 (43.2)	88 (56.8)	1.94 (1.2,3.12)	2.13 (1.3,3.5)
Female	149 (100)	42 (28.2)	107 (71.8)	1	1
**Educational level**					
1-4	131 (100)	47 (35.9)	84 (64.1)	1	-
5-8	173 (100)	62 (35.8)	111 (64.2)	0.99 (0.622,1.6)	
**Fathers’ occupation**					
Farmer	145 (100)	61 (42.1)	84 (57.9)	1.68 (1.05, 2.69)	1.96 (1.97,3.22)
Non-farmer	159 (100)	48 (30.2)	111 (69.8)	1	1
**Mothers’ occupation**					
House wife	190 (100)	68 (35.8)	122 (64.2)	0.93 (0.50,1.72)	
Government employee	28 (100)	8 (28.6)	20 (71.4)	0.67 (0.25,1.78)	-
Daily labourer	30 (100)	12 (40)	18 (60)	1.11 (0.45,2.76)	
Other	56 (100)	21 ()	35 ()	1	
**Fathers’ educational status**					
Does not read and write	66 (100)	23 (34.9)	43 (65.1)	2.0 (0.60,6.75)	
Able to read and write	165 (100)	65 (39.4)	100 (60.6)	2.44 (0.78,7.67)	-
Secondary school completed	54 (100)	17 (31.5)	37 (68.5)	1.72 (0.49,5.98)	
Expert level	19 (100)	4 (21.1)	15 (78.9)	1	
**Mothers’ educational status**					
Does not read and write	143 (100)	58 (40.6)	85 (59.4)	1.70 (0.63,4.66)	
Able to read and write	140 (100)	45 (32.1)	95 (67.9)	1.18 (0.43,3.26)	-
Secondary school completed	21 (100)	6 (28.6)	15 (71.4)	1	
**Swimming habit**					
Yes	274 (100)	100 (36.5)	174 (63.5)	1.34 (0.59,3.04)	
No	30 (100)	9 (30.0)	21 (70.0)	1	-
**Swimming regularity**					
Sometimes	214 (100)	79 (36.9)	135 (63.1)	1	-
Regularly	60 (100)	21 (35.0)	39 (65.0)	0.92 (0.51,1.68)	
**Living with biological parent**					
Yes	284 (100)	99 (34.9)	185 (65.1)	1	1
No	18 (100)	10 (55.6)	8 (44.5)	2.34 (0.89,6.12)	3.09 (1.14,8.4)
**Distance from river**					
<30min	213 (100)	74 (34.7)	139 (65.3)	0.85 (0.51,1.42)	-
>=30min	91 (100)	35 (38.5)	56 (61.2)	1	
**Water source for washing and bathing**					
Dam	91 (100)	35 (38.5)	56 (61.2)	0.85 (0.51,1.41)	-
other	213 (100)	74 (34.7)	139 (65.3)	1	
**Heard about urinary schistosomiasis**					
Yes	168 (100)	61 (36.3)	107 (63.7)	1	-
No	136 (100)	48 (35.3)	88 (65.7)	0.96 (0.59,1.53)	
**Total**	304 (100)	109 (35.9)	195 (64.1)

**Table 4:** **Multivariate analysis of variables associated with urinary schistosomiasis among study participants who had heard about urinary schistosomiasis, Abobo, southwestern Ethiopia, 2014**

**Variables**	**Frequency of urinary schistosomiasis**			**COR (95% CI)**	**AOR (95% CI)**
	**Sub-total**	**Positive (%)**	**Negative (%)**		
**Information source**					
School	76 (100)	27 (35.5)	49 (64.5)	1	
Home	46 (100)	15 (32.6)	31 (67.4)	0.88 (0.41,1.91)	-
Other	46 (100)	19 (41.3)	27 (58.7)	1.28 (0.60,2.71)	
**Symptom of urinary schistosomiasis**					
Abdominal pain	16 (100)	8 (50.0)	8 (50.0)	1	
Dysuria	5 (100)	1 (20.0)	4 (80.0)	0.25 (0.02,2.76)	
Bloody urine	121 (100)	44 (36.4)	77 (63.6)	0.57 (0.20,1.63)	-
Weakness	4 (100)	1 (25.0)	3 (75.0)	0.33 (0.03,3.93)	
Do not know	22 (100)	7 (31.8)	15 (68.2)	0.47 (0.12,1.77)	
**Parasite penetrate skin**					
Yes	48 (100)	18 (37.5)	30 (62.5)	1	-
No	120 (100)	43 (35.8)	77 (64.2)	0.93 (0.47,1.86)	
**Know the vector**					
Yes	30 (100)	14 (46.7)	16 (53.3)	1	-
No	138 (100)	47 (34.1)	91 (65.9)	0.59 (0.27, 1.31)	
**Have prevention**					
Yes	116 (100)	50 (43.1)	66 (56.9)	1	1
No	25 (100)	5 (20.0)	20 (80.0)	0.33 (0.12,0.94)	0.32 (0.11,0.96)
Do not know	27 (100)	6 (22.2)	21 (77.8)	0.38 (0.14,1.00)	0.29 (0.6,1.35)
**Have treatment**					
Yes	142 (100)	55 (38.7)	87 (61.3)	1	
No	10 (100)	2 (20.0)	8 (80.0)	0.40 (0.08,1.93)	-
Do not know	16 (100)	4 (25.0)	12 (75.0)	0.53 (0.16,1.74)	
**Total**	168 (100)	61 (36.3)	107 (63.7)		

AOR = adjusted odd ratio; indicated only for significant variables with p-value less than 0.05.

CI = confidence interval.

COR = crude odd ratio.

## Discussion

In the present study, the overall prevalence of urinary schistosomiasis among school children in Abobo area, Gambella, was 35.9% and can be categorized as moderate based on WHO categories of endemic communities [16]. This prevalence was higher compared to studies carried out in the Afar Region (24.54%), the middle Awash Valley (3.1%) and Somali Region (16.0%) [14,17,18] of Ethiopia. It was also higher compared to studies conducted in Sudan (16%) and Swaziland (5.3%) [19,20]. However, it was lower than a prevalence reported from Hassoba in Afar Regional state, Ethiopia (47.6%), from the White Nile River Basin of the Sudan (45%), and from Nigeria (41.5%) [21-23]. The difference can be explained by differences in ecological factors that can in turn lead to differences in transmission intensity [24].

This study showed lower mean intensity of urinary schistosomiasis (8.76 per 10 ml of urine) as compared to a study carried out in Afar Region, Ethiopia (14.8 eggs/10 ml of urine), Senegal (185eggs/10 ml of urine) and the Sudan (14.9 eggs/10 ml) [23,25,26]. This could be associated with differences in seasonality in transmission and types of water contact among study participants [24,25,27]. Treatment against urinary schistosomiasis could also lead to the low intensity of the parasite as indicated from a previous study in Mali [28]. In the current study, 84.5% (142) and 72.0% (121) of study participants knew about the presence of treatment against urinary schistosomiasis and reported bloody urine as a symptom of urinary schistosomiasis, respectively. This might have also caused self-reporting and hence seeking treatment for the infection [29].

In the current study, males were two times more likely to be infected with urinary schistosomiasis than females. This was comparable with studies conducted in the Sudan and Swaziland [19,20,26]. Socio-cultural factors where males are mostly engaged in water- contact activities like swimming and bathing, fishing, farming and watering cattle could lead to higher exposure among males. Significant association between urinary schistosomiasis and school children involved in farming and fishing were reported from Nigeria [30]. On the contrary, studies carried out in Ghana and Nigeria showed higher prevalence of urinary schistosomiasis among females than males [31,32].

Even though the prevalence of urinary schistosomiasis increased with age, there was no significant association between these variables. This finding was in line with studies conducted in the Somali Regional State, Ethiopia and the Sudan [17,26]. This could be explained by more involvement of older children in field activities and relatively higher risk of exposure to infection. Previous study from Nigeria indicated that children in the 10 to 14 years age group excrete large numbers of *S. haematobium* eggs [33].

Occupation of father was significantly associated with urinary schistosomiasis where children with farmer as father’s occupation were two times more likely to be infected than children with non-farmer as father’s occupation [AOR (95% CI): 1.96 (1.19, 3.22)]. This result agreed with studies from Sudan, Ghana and Nigeria [21,26,29] where children participated in field activities with their fathers. This shows lack of awareness towards risk of urinary schistosomiasis among fathers to make their children aware about the risk of urinary schistosomiasis. A protective role of the head of the family being literate and informed on urinary schistosomiasis was reported from an earlier study in Nigeria [33].

In addition, children not living with their parents were three times more likely to be infected with urinary schistosomiasis [AOR (95% CI): 3.09 (1.14, 8.40)] than those living with their parent. Similar result were reported from Nigeria [33]. Children living alone could perform any activities including field and water-based tasks as a source of income, and might have exposed themselves to infection. An earlier study from Yemen reported low level of income and unsafe source of water as a risk factor for urinary schistosomiasis among school children [34].

It is interesting to observe high prevalence of urinary schistosomiasis among children with the habit of swimming without significant association. Similar result were reported from an earlier study in Nigeria [21]. Despite the high prevalence of urinary schistosomiasis with the habit of swimming, children swimming regularly accounted for 21% while those swimming sometimes accounted for 79% of the current total prevalence of urinary schistosomiasis. This indicates that long duration of hours to water contact was considered as an important risk factor for exposure to urinary schistosomiasis rather than frequency of water contact [35].

## Conclusion

The present study area in Gambella Regional State, southwestern Ethiopia, represents moderate-risk community for urinary schistosomiasis. Sex, father’s occupation and living apart from parents were the determinant factors for the infection. Treatment of all school-age children and fishermen is required once every 2 years until the prevalence of infection falls below the level of public health importance. It is also recommended to complement the praziquantel treatment with supplementary measures such as provision of sanitation facilities and health education.

### Definition of variables

Swimming habit: child behaviour in recreational water contact.

Swimming regularity: frequent recreational water contact of children.

Living with parent: children living in the household of his or her biological parents for over three months.

Distance from the river: the space between children’s home and the river.

Heard about urinary schistosomiasis: information children have acquired about transmission, signs and symptoms, diagnosis and treatment of urinary schistosomiasis.

Symptom of urinary schistosomiasis: information children have acquired about clinical symptoms of urinary schistosomiasis.

Parasites penetrate skin: information children have acquired as to whether or not the schistosome parasites penetrates human skin during infection.

Know the intermediate host: information children have acquired about schistosome intermediate hosts.

Have prevention: information children have acquired about prevention and control measures of urinary schistosomiasis.

Have treatment: information whether or not children knew the availability of treatment options against urinary schistosomiasis.

## References

[1] Gryseels B, Polman K, Clerinx J, Kestens L (2006). Human schistosomiasis. Lancet.

[2] World Health Organization. Schistosomiasis. Fact sheet Number 115. Available: www.who.int/mediacentre/factsheets/fs115/en. Accessed. February 18, 2015.

[3] CDC 2011. The burden of schistosomiasis. Available: http://www.cdc.gov/globalhealth/ntd/diseases/schisto_burden.html. Accessed: 12 February 2015.

[4] National Travel Health Network and Centre (NaTHNaC). Schistosomiasis. Available: https://www.nathnac.org/pro/factsheets/schisto.htm. 2008. Accessed: 08 November 2014.

[5] van der Werf MJ, de Vlas SJ, Brooker S, Looman CW, Nagelkerke NJ, Habbema JD (2003). Quantification of clinical morbidity associated with schistosome infection in sub-Saharan Africa. Acta Trop.

[6] Stephenson L (1993). The impact of schistosomiasis on human nutrition. Parasitology.

[7] World Health Organization (2002). Prevention and control of schistosomiasis and soil transmitted helminthiasis. WHO Technical Report Series, No. 912.

[8] World Health Organization (2010). Working to overcome the global impact of neglected tropical disease: first WHO report on neglected tropical diseases.

[9] Jamison TD, Breman GJ, Measham RA, Alleyne G, Claeson M, Evans BD (2006). Disease Control Priorities Project: Disease Control Priorities in Developing Countries.

[10] Deribe K, Eldaw A, Hadziabduli S, Kailie E, Omer MD, Mohammed AE (2011). High prevalence of urinary schistosomiasis in two communities in South Darfur: implication for interventions. Parasit Vectors.

[11] Bala AY, Ladan MU, Mainasara M (2012). Prevalence and intensity of urinary schistosomiasis in Abarma village, Gusau, Nigeria: a preliminary investigation. Sci World J.

[12] Kassa L, Omer A, Tafesse W, Taye T, Kebebew F, Beker A. Schistosomiasis: Diploma program for the Ethiopian health center team. Ethiopia public health training initiative. 2005:8–18.

[13] Ethiopian Health and Nutritional Research Institution. Proceeding of the international stakeholders’ consultative meeting on schistosomiasis and soli transmitted helminthes (STH) in Ethiopia. International workshop proceeding on schistosomiasis/STH. 2012:29–48.

[14] Deribew K, Tekeste Z, Petros B (2013). Urinary schistosomiasis and malaria associated anemia in Ethiopia. Asian Pac J Trop Biomed.

[15] World Health Organization (1991). Basic Laboratory Methods in Medical Parasitology.

[16] World Health Organization (2006). Preventive Chemotherapy in Human Helminthiasis: Coordinated Use of Anthelminthic Drugs in Control Interventions: A Manual for Health Professionals and Programme Managers.

[17] Negussu N, Wali M, Ejigu M, Debebe F, Aden S, Abdi R (2013). Prevalence and distribution of schistosomiasis in Afder and Gode zone of Somali region, Ethiopia. J Glob Infect Dis.

[18] Jemaneh L, Tedla S, Birrie H (1994). The use of reagent strips for detection of urinary schistosomiasis infection in the middle Awash Valley, Ethiopia. East Afr Med J.

[19] Dahab TO, El-Bingawi HM (2012). Epidemiological survey: *Schistosoma haematobium* in schoolchildren of White Nile areas, Khartoum. Sudan Med J.

[20] Liao CW, Sukati H, Nara T, Tsubouchi A, Chou CM, Jian JY (2011). Prevalence of Schistosoma haematobium infection among schoolchildren in remote areas devoid of sanitation in northwestern Swaziland, Southern Africa. Jpn J Infect Dis.

[21] Ayele B, Erko B, Legesse M, Hailu A, Medhin G (2008). Evaluation of Circulating Cathodic Antigen (CCA) strip for diagnosis of urinary schistosomiasis in Hassoba school children, Afar, Ethiopia. Parasite.

[22] Ismail H, Hong ST, Babiker A, Hassan R, Sulaiman M, Jeong HG (2014). Prevalence, risk factors, and clinical manifestations of schistosomiasis among school children in the White Nile River basin, Sudan. Parasit Vectors.

[23] Houmsou RS, Amuta EU, Sar TT (2012). Profile of an epidemiological study of urinary schistosomiasis in two local government areas of Benue state, Nigeria. Int J Biomed Res.

[24] Firth S, Dembele R, Garba A, Toure S, Sacko M, Landoure A, Bosque-Oliva E, Barnett AG, Brooker S, Fenwick A, Clements ACA (2009). Use of bayesian geostatistical prediction to estimate local variations in *Schistosoma haematobium* infection in western Africa. Bull World Health Organ.

[25] Senghor B, Diallo A, Sylla SN, Doucoure S, Ndiath MO, Gaayeb L (2014). Prevalence and intensity of urinary schistosomiasis among school children in the district of Niakhar, region of Fatick, Senegal. Parasit Vectors.

[26] Abou-Zeid AH, Abkar TA, Mohamed RO (2013). Schistosomiasis infection among primary school students in a war zone, Southern Kordofan State, Sudan: a cross-sectional study. BMC Public Health.

[27] Ivoke N, Ivoke ON, Nwani CD, Ekeh FN, Asogwa CN, Atama CI (2014). Prevalence and transmission dynamics of *Schistosoma haematobium* infection in a rural community of southwestern Ebonyi State, Nigeria. Trop Biomed.

[28] Landoure A, Dembele R, Goita S, Kane M, Tuinsma M, Sacko M (2012). Significantly reduced intensity of infection but persistent prevalence of schistosomiasis in a highly endemic region in Mali after repeated treatment. PLoS Negl Trop Dis.

[29] Lengeler C, Utzinger J, Tanner M (2002). Questionnaires for rapid screening of schistosomiasis in sub-Saharan Africa. Bull World Health Organ.

[30] Risikat SA, Ayoade AA (2012). Correlation analysis between the prevalence of *Schistosoma haematobuim* and water conditions: A Case Study among the School Pupils in Southwestern Nigeria. IJRRAS.

[31] Gyuse KI, Ofoezie IE, Ogunniyi TAB (2010). The effect of urinary schistosomiasis on the health of children in selected rural communities of Osun State, Nigeria. J Trop Med Parasitol.

[32] Okanla EO, Agba BN, Awotunde JO (2003). *Schistosoma haematobium*: prevalence and socio-economic factors among students in Cape Coast Ghana. Afr J Biomed Res.

[33] Ugbomoiko US, Ofoezie IE, Okoye IC, Heukelbach J (2010). Factors associated with urinary schistosomiasis in two peri-urban communities in south–western Nigeria. Ann Trop Med Parasitol.

[34] Sady H, Al-Mekhlafi HM, Mahdy MA, Lim YAL, Mahmud R, Surin J (2013). Prevalence and associated factors of schistosomiasis among children in Yemen: Implications for an effective control programme. PLoS Negl Trop Dis.

[35] Hassan AO, Amoo AOJ, Akinwale OP, Deji-Agboola AM, Adeleke MA, Gyang PV (2012). Human water contact activities and urinary schistosomiasis around Erinle and Eko-ende dams. Global Adv Res J Med Med Sci.

